# Safety and Treatment Outcomes of Nivolumab for the Treatment of Recurrent or Metastatic Head and Neck Squamous Cell Carcinoma: Retrospective Multicenter Cohort Study

**DOI:** 10.3390/cancers13061413

**Published:** 2021-03-19

**Authors:** Ifigenia Vasiliadou, Omar Breik, Holly Baker, Isla Leslie, Van Ren Sim, Gemma Hegarty, Andriana Michaelidou, Kannon Nathan, Andrew Hartley, James Good, Paul Sanghera, Charles Fong, Teresa Guerrero Urbano, Mary Lei, Imran Petkar, Miguel Reis Ferreira, Chris Nutting, Kee Howe Wong, Kate Newbold, Kevin Harrington, Shree Bhide, Anthony Kong

**Affiliations:** 1 Guys Cancer Centre, Guy’s and St. Thomas NHS Foundation Trust, London SE1 9RT, UK; ifigenia.vasiliadou@nhs.net (I.V.); nernav@gmail.com (V.R.S.); teresa.guerrero-urbano@kcl.ac.uk (T.G.U.); Mary.Lei@gstt.nhs.uk (M.L.); Imran.Petkar@gstt.nhs.uk (I.P.); Miguel.ReisFerreira@gstt.nhs.uk (M.R.F.); 2 Department of Oncology, Queen Elizabeth Hospital Birmingham, Birmingham B15 2WB, UK; omar.breik@gmail.com (O.B.); HCB497@student.bham.ac.uk (H.B.); Andrew.Hartley@uhb.nhs.uk (A.H.); James.Good@uhb.nhs.uk (J.G.); Paul.Sanghera@uhb.nhs.uk (P.S.); Charles.Fong@uhb.nhs.uk (C.F.); 3 Department of Oral and Maxillofacial Surgery, Royal Brisbane and Women’s Hospital, QLD 4029 Brisbane, Australia; 4 College of Medical and Dental Sciences, University of Birmingham, Birmingham B15 2TT, UK; 5 Head and Neck Unit, Royal Marsden NHS Foundation Trust, London SW3 6JJ, UK; Isla.Leslie@rmh.nhs.uk (I.L.); Chris.Nutting@rmh.nhs.uk (C.N.); KeeHowe.Wong@rmh.nhs.uk (K.H.W.); Kate.Newbold@rmh.nhs.uk (K.N.); Kevin.Harrington@icr.ac.uk (K.H.); Shree.Bhide@icr.ac.uk (S.B.); 6 Kent Oncology Centre, Maidstone and Tunbridge Wells NHS Trust, Kent ME16 9QQ, UK; gemmahegarty@nhs.net (G.H.); a.michaelidou@nhs.net (A.M.); Kannon.nathan@nhs.net (K.N.); 7 Division of Radiotherapy and Imaging, The Institute of Cancer Research, London SW7 3RP, UK; 8 Comprehensive Cancer Centre, King’s College London, Guy’s Campus, London SE1 1UL, UK

**Keywords:** nivolumab, immunotherapy, head and neck squamous cell carcinoma, recurrent/metastatic HNSCC, immune-related toxicities, real-world data, palliative

## Abstract

**Simple Summary:**

Nivolumab is an anti-PD-1 monoclonal antibody that has been approved in the management of recurrent/metastatic head and neck squamous cell carcinoma (HNSCC) with evidence of progression within 6 months of platinum-based chemotherapy. Most of the evidence regarding the use of nivolumab in this setting is provided within clinical trials with strict inclusion criteria. This multicenter retrospective cohort study evaluates the real-world outcomes of nivolumab use in four major cancer centers in England. This study demonstrates similar progression free survival and overall survival as that seen in the CHECKMATE-141 study. In addition, it demonstrates that nivolumab tends to be well tolerated, with only a 15% rate of immune-related toxicity. Our findings support the findings of other real-world studies that demonstrate improved progression free survival and potentially overall survival for those who have immune-related toxicity.

**Abstract:**

Nivolumab is an anti-PD-1 monoclonal antibody currently used as immunotherapy for patients with recurrent/metastatic head and neck squamous cell carcinoma (HNSCC) with evidence of disease progression after platinum-based chemotherapy. This study evaluates real-world safety and treatment outcomes of non-trial nivolumab use. A retrospective multicenter cohort study of patients with recurrent/metastatic HNSCC treated with nivolumab between January 2017 and March 2020 was performed. Overall, 123 patients were included. The median age was 64 years, the majority of patients were male (80.5%) and had a smoking history (69.9%). Primary outcomes included overall response rate (ORR) of 19.3%, median progression-free survival (PFS) of 3.9 months, 1-year PFS rate of 16.8%, a median overall survival (OS) of 6.5 months and 1-year OS rate of 28.6%. These results are comparable to the CHECKMATE-141 study. Of 27 patients who had PD-L1 status tested, positive PD-L1 status did not significantly affect PFS (*p* = 0.86) or OS (*p* = 0.84). Nivolumab was well tolerated with only 15.1% experiencing immune-related toxicities (IRT) and only 6.7% of patients stopping due to toxicity. The occurrence of IRT appeared to significantly affect PFS (*p* = 0.01) but not OS (*p* = 0.07). Nivolumab in recurrent/metastatic HNSCC is well tolerated and may be more efficacious in patients who develop IRT.

## 1. Introduction

Head and neck cancers are the sixth most common cancers worldwide, and are the eighth most common cause of cancer-related death [1]. The most common histology of head and neck cancers is squamous cell carcinoma (SCC), collectively known as head and neck squamous cell carcinomas (HNSCC), accounting for 90% of all cases. Most HNSCCs are associated with smoking and alcohol intake but there is increasing incidence of oropharyngeal cancer due to human papillomavirus (HPV) infection [2].

Around 20–40% of patients with HNSCC will suffer local/regional recurrence or metastatic disease after completing primary treatment consisting of a combination of surgery, radiotherapy and/or chemotherapy [3,4,5]. Few of these patients may be salvaged with surgery or re-irradiation. The majority of these patients will only be suitable for palliative treatments and supportive care, with a poor prognosis and a median overall survival of less than 1 year. Until recently, first-line systemic treatment for recurrent or metastatic HNSCC patients has been based on the EXTREME regimen, a platinum-based chemotherapy (cisplatin or carboplatin and fluorouracil) with cetuximab, an IgG1 chimeric monoclonal antibody to epidermal growth factor receptor (EGFR) [6]. Cetuximab in the palliative setting is only approved for oral cavity SCC in the UK. The addition of cetuximab to platinum-based chemotherapy improved the median overall survival (OS) from 7.4 months to 10.1 months and progression-free survival (PFS) from 3.3 to 5.6 months [6].

Nivolumab, a fully human IgG4 anti-PD-1 monoclonal antibody has demonstrated efficacy against a variety of tumor types [7,8,9,10,11,12]. The pivotal study that established the role of nivolumab in HNSCC was CheckMate-141, a randomized controlled trial (RCT) comparing nivolumab with investigator’s choice of standard of care therapies (docetaxel, methotrexate or cetuximab) in 361 patients with platinum-refractory recurrent/metastatic HNSCC [13]. Patients treated with nivolumab had better OS than those receiving standard therapy (median OS 7.5 months vs 5.1 months) although there was no increase in median PFS (2 months vs 2.3 months with standard of care) and objective response rate (ORR) remained poor (13.3% in the nivolumab group versus 5.8% in the standard of care) [13]. In addition, nivolumab demonstrated a better toxicity profile compared to standard therapy [13]. Based on these findings, nivolumab was approved by the Food and Drug Administration (FDA) in November 2016, and the European Medicines Agency (EMA) in April 2017 as second-line therapy for platinum refractory disease. On 22 November 2017, the National Institute for Health and Care Excellence (NICE) approved nivolumab for recurrent/metastatic HNSCC patients through the NHS England Cancer Drugs Fund (CDF) if they have progressed within 6 months of having platinum-based chemotherapy, either as neoadjuvant treatment, adjuvant treatment or in the palliative setting [14]. The CDF provides funding for promising cancer drugs in England to ensure value for money for taxpayers and to offer a fast-track route to NHS funding for pharmaceutical companies [15].

In addition, pembrolizumab was recently approved worldwide for first-line treatment for recurrent/metastatic HNSCC based on KEYNOTE-048 trial data, which showed that pembrolizumab extended overall survival compared to the EXTREME chemotherapy regime in patients with HNSCC tumors with combined positive score (CPS) >20 (HR = 0.61; *p* = 0.0007) and >1 (HR = 0.78; *p* = 0.0086) [16]. Furthermore, pembrolizumab plus cisplatin and fluorouracil chemotherapy had superior OS compared to the EXTREME regimen in the PD-L1 CPS ≥ 20, CPS ≥ 1, and total populations with comparable safety.

Outside of clinical trials, few studies have provided real-world data on the clinical outcomes of nivolumab since its approval [17,18,19,20]. There has been an increased interest in routinely collected data to validate the findings of RCTs, and to generate evidence on challenges and treatment effects in the real clinical world [21]. In addition, it is known that the randomized trial Checkmate-141 had strict eligibility criteria including exclusion of non-SCC and paranasal SCC patients, which may affect its applicability in the real world. The aim of this study was to provide multicenter real-world data on the efficacy and treatment outcome from the use of nivolumab in metastatic/recurrent HNSCC prior to the approval of pembrolizumab in the UK. Our objectives were to collect the characteristics of tumors and patients treated with nivolumab for recurrent/metastatic HNSCC and to assess the evidence of clinical benefit of nivolumab as well as to collect data on immune-related toxicities.

## 2. Materials and Methods

This study was a retrospective data analysis of patients with recurrent/metastatic HNSCC treated with nivolumab as second-line treatment at four cancer centers across the UK (Queen Elizabeth Hospital (QEH), Birmingham, Guy’s Cancer Centre, London, Kent Oncology Centre, Maidstone, and Royal Marsden Hospital, London and Sutton) from January 2017 to March 2020 before the introduction of pembrolizumab as first-line treatment during the Covid-19 pandemic (Covid-19 interim approval by NHS England was first introduced in March 2020 followed by CDF approval on 25 November 2020). We included patients treated with nivolumab after the approval of nivolumab under CDF by NICE on 22 November 2017. To capture the whole picture of nivolumab use in the real world, we also included those patients who had nivolumab outside of CDF (self-payment through co-payment policy before and after CDF approval) or those who had CDF approval for nivolumab but did not receive it due to rapid disease progression. Patients were excluded from the study if they were administered nivolumab within a trial, or if they were referred for a cutaneous malignancy and patients were also excluded from the efficacy analysis if the tumors were not mucosal HNSCCs.

The collected patient data included baseline characteristics, details of treatments, response to treatment, survival data, and treatment-related toxicities. All information was obtained retrospectively from the electronic patient records.

### 2.1. Patient and Tumor Characteristics

The baseline characteristics included age, gender, past medical history, medications (specifically the use of oral steroids at baseline before the commencement of anti-PD1 antibody), smoking history, and alcohol history. The tumor characteristics include primary tumor subsite (if known), date of initial diagnosis, staging at initial diagnosis, and PD-L1 status (if performed). PD-L1 testing was done using immunohistochemistry (IHC) with the use of a rabbit antihuman PD-L1 antibody (clone 28–8, Epitomics) as per Dako protocol and was defined as positive if scored ≥1 in a minimum of 100 tumor cells [13]. The clinical staging at diagnosis was classified based on the 7th edition of the TNM classification [22].

### 2.2. Treatment Characteristics and Survival Data

The treatment characteristics included intent of treatment (radical versus palliative) at diagnosis, type of primary treatment, date of diagnosis of recurrent/metastatic disease, previous treatment of recurrent/metastatic disease, previous use of cetuximab, and duration of treatment with immunotherapy.

Objective response rate (ORR) as assessed by radiologists was collected from electronic patients’ records. PFS was defined as the time from immunotherapy commencement date to the date of disease progression or death. Progression of disease was confirmed by imaging and/or clinically. OS was defined as the time from immunotherapy commencement date to the date of death from any cause. Treatment toxicities were graded according to the NCI Common Terminology Criteria for Adverse Events, version 4.0 [23].

### 2.3. Statistical Analysis

Statistical analysis was performed using the Statistical Package for the Social Sciences (SPSS) version 26. The data cutoff point for the analysis was 30 May 2020. The distribution of PFS and OS was estimated by the Kaplan–Meier method and compared by using the log-rank test. Results with *p*-value of less than 0.05 were regarded as statistically significant.

## 3. Results

### 3.1. Patient and Tumor Characteristics

From January 2017 to March 2020, 123 patients were offered nivolumab as the standard of care treatment (including 3 patients who paid for nivolumab under co-payment policy, 1 patient under compassionate access, as well as 3 patients who subsequently did not receive the treatment due to rapid disease progression). The patient characteristics are summarized in Table 1. The median age at the commencement of nivolumab was 64 years. The majority of the patients were male (80.5%) and had a smoking history, either as an ex-smoker or current smoker (69.9%). Only 3 patients (2.4%) were on steroids prior to commencement of immunotherapy; however, there was no detailed recording of dose and type of oral steroid, the commencement date, and reasons for starting steroids.

All patients had the histological diagnosis of squamous cell carcinoma apart from one patient who had Epstein–Barr virus-related undifferentiated carcinoma of the nasopharynx who paid for nivolumab under the co-payment policy while remaining under the NHS care. This patient has been removed from the final analysis of outcomes since the tumor was not squamous cell carcinoma. Primary tumor sites included oral cavity (*n*= 33; 26.8%), pharynx (nasopharynx, oropharynx, and hypopharynx) (*n*= 52; 42.3%), larynx (*n*= 25; 20.3%), paranasal sinuses (*n* = 5; 4.1%), and unknown primary (*n* = 8; 6.5%). At time of diagnosis, the most common primary site was the oropharynx (*n* = 43; 35.0%) and most of the patients had stage 4 disease according to TNM7 (*n* = 97; 78.9%). Of the 123 HNSCC patients, 38.2% (*n* = 47) had loco-regional recurrence, 13.0% (*n* = 16) had local recurrence and metastatic disease, and 28.5% (*n* = 35) had metastatic disease with no evidence of locoregional recurrence. Twenty-eight (22.8%) patients received treatment with palliative intent at diagnosis for either advanced or metastatic disease due to non-suitability for curative radical treatment. Only 23.6% had the PD-L1 status tested (*n* = 29); 41.4% of these tested tumors were classed as positive (PD-L1 ≥ 1%) (Table 1).

### 3.2. Treatment Characteristics

Seventy-seven percent (*n* = 95; 77.2%) of the patients were treated with curative intent at primary diagnosis. Thirty-one percent (*n* = 38; 30.9%) received primary surgery and forty-nine percent (*n* = 59; 49.0%) primary or adjuvant radiotherapy (+/- concurrent or induction chemotherapy). Seventeen percent (*n* = 21; 17.1%) received palliative chemotherapy and four percent (*n* = 5; 4.1%) palliative radiotherapy at presentation (Table 2).

**Figure 1:**
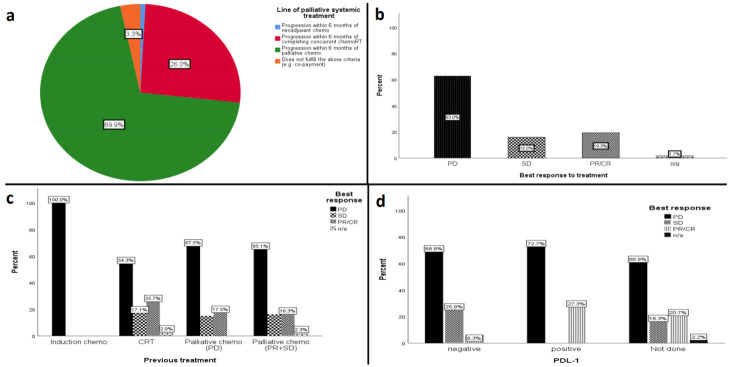
(**a**) Line of palliative systemic treatment. Best response to treatment for (**b**) all patients; (**c**) based on previous treatment; (**d**) based on PD-L1 status.

Patients were planned to receive or received nivolumab treatment as follows (Figure 1a):

(1) CDF: Nivolumab following recurrence or progression after neo-adjuvant chemotherapy (*n* = 1); within 6 months of completing primary/adjuvant concurrent chemoradiotherapy with platinum-based chemotherapy (*n* = 32); within 6 months of receiving palliative chemotherapy with platinum-based chemotherapy (*n* = 86).
(2) Four patients did not fit the criteria above (*n* = 2, co-payment: one before CDF approval and the other had nasopharynx cancer; 1 compassionate access; 1 received nivolumab having progressed more than 6 months after platinum chemotherapy).

### 3.3. Efficacy

We excluded 4 patients from the efficacy and safety analysis (1 patient with Epstein Barr Virus (EBV)associated nasopharyngeal undifferentiated carcinoma and 3 patients who did not commence treatment due to rapid deterioration). The objective response rate (ORR) was 19.3% (*n* = 23): 4 patients with a complete response (CR) and 19 patients with a partial response (PR) to treatment (Table 3 and Figure 1b). 16.0% (*n* = 19) had stable disease and 63.0% (*n* = 75) had progressive disease. We further categorized response to nivolumab based on previous cancer treatment. Only one patient received nivolumab with progressive disease after neoadjuvant chemotherapy (100%); 25.7% of patients had CR/PR to nivolumab treatment following concurrent chemoradiotherapy. 16.3% and 17.5% of patients had CR/PR to nivolumab treatment following a previous response or non-response to palliative platinum chemotherapy, respectively (Figure 1c).

### 3.4. PD-L1 Express PD-L1 Expression Status and Response to Treatment

PD-L1 expression status was evaluated in 27 out of 119 patients (22.6%) who received nivolumab. Despite the CDF form requiring confirmation that every effort had been made for the patient to have PD-L1 testing, 77.4% of these patients did not have their PD-L1 status recorded or test performed. This could be because the test is not routinely done as part of the standard of care in many hospitals and the result is not required prior to electronic CDF approval. Among the patients evaluated, 11 patients (40.7%) had a PD-L1 expression of ≥ 1. 27.3% (3/11) of PD-L1 positive patients had CR/PR as the best response to treatment compared to 6.3% (1/16) of PD-L1 negative patients (Figure 1d).

### 3.5. Progression-Free Survival (PFS)

**Figure A1:**
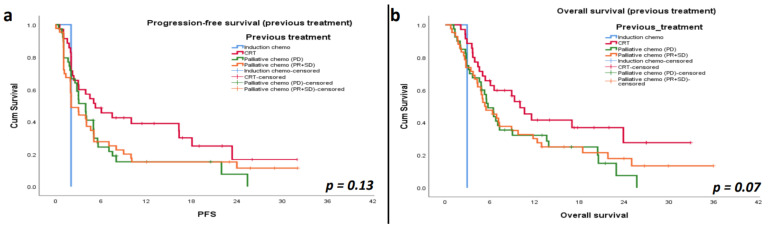
Kaplan–Meier curves for (**a**) progression-free survival and (**b**) overall survival in patients in the nivolumab group based on previous treatment.

**Figure A2:**
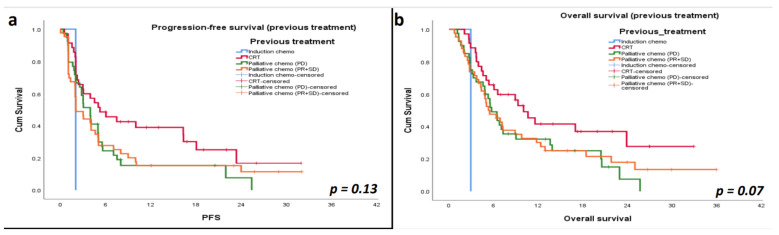
Kaplan–Meier curves for (**a**) progression-free survival and (**b**) overall survival in patients in the nivolumab group based on PD-L1 status.

At the time of analysis, 98 patients (82.4%) had progressed from the total of 119 patients treated with nivolumab and included in the analysis. The median PFS was 3.9 months (95% confidence interval (CI) 3.1–4.8 months). The estimated rate of PFS at 1 year was 16.8%. In the subgroup analysis of PFS according to primary treatment for the nivolumab-treated patients, the median PFS for the chemoradiotherapy group was 5.2 months (95% CI 1.2–9.2 months) compared to 3.9 months (95% CI 2.4–5.5 months) for non-responders to palliative chemotherapy and 2.0 months (95% CI 0.7–3.3 months) for responders to palliative chemotherapy but the differences were statistically non-significant (*p* = 0.13; Appendix A Figure A1a). We also did not detect any difference in the median PFS between PD-L1 positive (3.9 months; 95% CI 2.3–5.6) and PD-L1 negative patients (3.9 months; 95% CI 2.1–5.8) among those treated with nivolumab, although the numbers were small (*p* = 0.86; Appendix A Figure A2a).

### 3.6. Overall Survival (OS)

**Figure 2:**
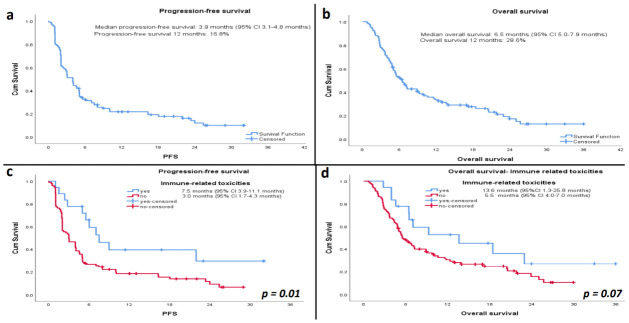
Kaplan–Meier curves for (**a**) progression-free survival and (**b**) overall survival. Kaplan–Meier curves for (**c**) progression-free survival and (**d**) overall survival in patients with or without immune-related toxicities (IRTs).

At the time of analysis, 89 deaths (74.8%) occurred in the total of 119 patients treated with nivolumab and included in the analysis. The median OS was 6.5 months (95% CI 5.0–7.9 months) (Figure 2b). The estimated rate of OS at 1 year was 28.6% (*n* = 34). In the subgroup analysis of OS according to primary treatment, the median OS for chemoradiotherapy group was 10.0 months (95% CI 6.9–13.1 months) compared to 5.7 months (95% CI 4.3–7.1 months) for non-responders of palliative chemotherapy and 5.3 months (95% CI 3.3–7.2 months) for responders to palliative chemotherapy but the differences were not statistically significant (*p* = 0.07; Appendix A Figure A1b). We also did not detect differences in median OS between PD-L1 positive (5.5 months; 95% CI 3.4–7.6 months) and PD-L1 negative patients (6.3 months; 95% CI 4.7–8.0 months) (log-rank test *p* = 0.84; Appendix A Figure A2b).

### 3.7. Reasons for Stopping Treatment

In total 70.6% patients had stopped treatment due to progressive disease (PD) and/or death. At the time of analysis, 5.0% of patients were actively having ongoing treatment. About two percent of patients had completed 24 months of nivolumab treatment. Toxicities determined treatment discontinuation in 6.7% of patients (Table 3).

### 3.8. Safety and Immune-Related Toxicity (IRT)

Immune-related toxicities (IRT) associated with nivolumab treatment are shown in Table 4. In 119 patients included in the analysis, we noted 18 (15.1%) immune-related toxicities that were documented in the electronic record. Pneumonitis, hepatitis, colitis, and endocrine toxicities were the most common immunotherapy-related toxicities. Other documented immune-related toxicities include lichen planus, hyponatremia, and thrombocytopenia. Eight patients stopped the treatment due to immune-related toxicities from treatment. There were no recorded deaths attributed to treatment toxicity. The PFS was 7.5 months in the IRT group (95% CI 3.9–11.1 months) compared to 3.0 months in the non-IRT group (95% CI 1.7–4.4 months), which was statistically significant (*p* = 0.01) (Figure 2c). The OS was 13.6 months in the IRT group (95% CI 1.4–25.8 months) compared to 5.5 months in the non-IRT group (95% CI 4.0–7.0 months), which was not statistically significant (*p* = 0.07) (Figure 2d). The steroids’ use for the management of immune-related toxicity was not collected. The overall response rate was statistically significant higher in the IRT group (7/17;41.2%) compared to the non-IRT group (16/100; 16.0%) (χ^2^ = 0.016).

## 4. Discussion

In this retrospective study we have collated the clinical experience of the use of nivolumab with outcome data for the treatment of recurrent/metastatic HNSCC as standard of care at four cancer centers in England.

The final analysis of 119 patients receiving nivolumab showed an ORR of 19.3%, a median PFS of 3.9 months, 1-year PFS rate of 16.8%, a median OS of 6.5 months and 1-year OS rate of 28.6%. These results are comparable to the results of Checkmate-141. The efficacy and safety of the use of nivolumab in this setting has been validated by other retrospective studies completed in Japan which showed an ORR ranging from 13.5%–29.6% and a median PFS of 3.7 months to 25 weeks [17,19,20].

The difference in PFS in this study compared to CHECKMATE-141 (3.9 months vs 2 months) can be partly explained by the delay in completing a response scan in real-world data compared to an earlier time-scheduled scan as part of a trial. The OS was very similar compared to the results of CHECKMATE-141 (6.5 months vs 7.5 months) [13].

The CHECKMATE-141 study showed that nivolumab improved OS compared to the investigator’s choice of cytotoxic therapy (docetaxel, methotrexate or cetuximab) and greater survival benefit was seen in the subgroup of PD-L1 expression ≥1 (the hazard ratio for death 0.55 (95% CI, 0.36 to 0.83)) compared to the subgroup of PD-L1 <1 (hazard ratio 0.89 (95% CI, 0.54 to 1.45)) [13,24,25]. In our cohort, only 22.7% of patients (*n* = 27) were tested for PD-L1 status. Bearing this limitation in mind, 27.3% (3/11) of PD-L1 positive patients had CR/PR as the best response to treatment while only 6.3% (1/16) of PD-L1 negative patients had CR/PR as the best response to treatment. Despite this apparent trend towards a better response in PD-L1 positive patients, ultimately our findings demonstrated no statistically significant difference in either PFS or OS in the PD-L1 expressors and non-expressors, which is likely due to the small number of patients (*n* = 27) included in the analysis. Thus, further studies with larger patient numbers are needed to confirm these findings.

These data demonstrate that nivolumab is generally well tolerated by patients, with only 15.1% of patients experiencing clinically-relevant toxicities, and 6.7% discontinuing treatment due to their development. The majority of these toxicities were deemed to be immune-related including pneumonitis, hepatitis, immune-related endocrinopathies or skin eruptions. Immune-related adverse events or toxicities (IRT) have been shown to be associated with higher anti-tumor responses and a clinical benefit in non-small cell lung carcinoma, renal cell carcinoma, and melanoma patients [18]. In two recent studies including head and neck patients, the development of IRT was associated with a better clinical outcome including 1-year survival rate [17] and OR rate [19]. Our findings further support this observation, as both median OS and PFS were higher in the group who experienced immune-related toxicity compared to the group with no toxicity (PFS was statistically significant higher in the immune-related toxicity group compared to the no-toxicity group but not OS).

Although this study evaluates the role of nivolumab in recurrent/metastatic HNSCC after progression on previous platinum chemotherapy, other studies such as Keynote 048 trial have demonstrated the potential benefits of receiving immunotherapy prior to or concurrently with platinum-based chemotherapy [16]. Other ongoing studies are evaluating the use of immunotherapy in combination with standard treatments in the curative or adjuvant setting. Interestingly, although the addition of pembrolizumab as first-line treatment for recurrent/metastatic HNSCC has shown significant survival advantage, Keynote 048 trial did not demonstrate that pembrolizumab had any advantage in PFS over chemotherapy [16]. However, the analysis of PFS after subsequent lines of treatment has shown a longer median PFS (defined as time from initiation of first line therapy to objective tumor progression or death from any cause) after the next line of therapy for those receiving pembrolizumab as first-line compared to those who received chemotherapy as first-line [26]. This highlights the importance of choosing the optimal timing of the use of immunotherapy in this setting. It may be that an initial exposure to an immune checkpoint inhibitor can cause changes to the tumor microenvironment and change the interaction of the immune system with tumor cells in the long-term, affecting response to subsequent lines of treatment [26]. Studies such as KEYNOTE-689 evaluating the role of pembrolizumab as neoadjuvant treatment and subsequently adjuvant treatment with standard of care in curative treatment of head and neck cancers may help to demonstrate the effect of pembrolizumab on tumor microenvironment and subsequent therapies, albeit in a curative setting [27]. Real-world data using pembrolizumab will need to be collected and analyzed to confirm efficacy and safety of the treatment in this setting if this is approved in the curative or adjuvant setting in the future.

The use of immunotherapy has been established both in the adjuvant and metastatic setting for a number of malignancies [9,10,11,12]. However, the development of predictive biomarkers is important in preventing unnecessary toxicities in the group of patients that are unlikely to respond, as well as guiding clinical decision-making in regard to the choice of order of therapies to achieve optimal response. A number of different variables have been associated with the prediction of the response to treatment such as PD-L1 expression, tumor-mutational burden (TMB), tumor-infiltrating lymphocytes (TILs) within a tumor and peripheral blood neutrophil to lymphocyte ratio value and gut microbiome [28,29]. Moreover, recent evidence showed that in patients with non-small cell lung cancer who received PD-(L)1 treatment, the use of baseline corticosteroid of ≥10 mg of prednisone equivalent was associated with poorer outcome in both PFS and OS whereas corticosteroids used for the management of immune-related adverse events have not affected efficacy [30]. This group of patients is largely excluded from clinical trials and real-world data are required to further investigate the effect of baseline corticosteroids. Only a small number of patients have been on baseline steroids in our group and the information was poorly documented; thus, we could not use it for further analysis [30]. Therefore, further research is vital in identifying potential biomarkers for response to immunotherapy in recurrent/malignant HNSCC.

## 5. Conclusions

In this report, we have demonstrated the efficacy of nivolumab in a real-world setting at several cancer centres in England. The most important limitations of this study are the retrospective nature of the study, the small numbers of reported PD-L1 status, and potential inadequate recording of minor non-immune-related adverse events. Despite these limitations, the findings of this multicenter retrospective series add to the data on the use of nivolumab in real-life settings and support both the results of CHECKMATE-141 and subsequent real-world studies investigating the efficacy and safety of nivolumab use in recurrent/metastatic HNSCC patients. Our results also suggest that patients who developed immune-related toxicity (IRT) seemed to have a better PFS compared to those who did not. Further research is needed to determine factors predicting response to immunotherapy in order to better select appropriate patients who will benefit from therapy and to determine the ideal timing for treatment to achieve maximum benefit.

## Figures and Tables

**Table 1:** Baseline tumor and patient characteristics.

Characteristics	Nivolumab Patients (*n* = 123)
Age (years)	
Median (range)	64 (22–94)
Male sex (%)	99 (80.5%)
Smoking status (%)	
Never	23 (18.7%)
Ex-smoker	48 (39.0%)
Current smoker	38 (30.9%)
Not recorded	14 (11.4%)
Site of primary tumor (%)	
Oral cavity	33 (26.8%)
Nasopharynx	1 (0.8%)
Oropharynx	43 (35.0%)
Hypopharynx	8 (6.5%)
Larynx	25 (20.3%)
Paranasal sinuses	5 (4.1%)
Unknown primary	8 (6.5%)
Staging TNM7 at time of diagnosis	
1	2 (1.6%)
2	5 (4.1%)
3	17 (13.8%)
4	
4a	75 (61.0%)
4b	7 (5.7%)
4c	15 (12.2%)
Not available	2 (1.6%)
PD-L1 status (%)	
Negative	17 (13.8%)
≥1%	12 (9.8%)
Not tested	94 (76.4%)

**Table 2:** Treatment characteristics.

Characteristics	Nivolumab Patients (*n* = 123)
Intent of treatment at diagnosis (%)	
Curative	95 (77.2%)
Palliative	28 (22.8%)
Primary treatment (%)	
Surgery +/− adjuvant (C)RT (including IC)	38 (30.9%)
(C)RT +/− IC	59 (48.0%)
Palliative chemotherapy	21 (17.1%)
Palliative radiotherapy	5 (4.0%)

**Table 3:** Treatment response and reasons for stopping treatment.

Characteristics	Nivolumab Patients (*n* = 119)
Best response to treatment (%)	
PD	75 (63.0%)
CR/PR	23 (19.3%)
SD	19 (16.0%)
Ν/A	2 (1.7%)
Reason for stopping treatment (%)	
PD	74 (62.2%)
Death	10 (8.4%)
Toxicity	8 (6.7%)
Ongoing treatment	6 (5.0%)
Not fit for treatment	11 (9.3%)
Completion of treatment (24 months)	3 (2.5%)
Patient choice	1 (0.9%)
n/a	6 (5.0%)

**Table 4:** Immune-related toxicities of interest.

IO-Related Toxicities	Total = 18
Pneumonitis	*n* = 3; Grade 3 (1), suspected pneumonitis (2)
Hepatitis	*n* = 3; Grade 4 (1), grade 3 (1), grade 2 (1)
Colitis	*n* = 3; Grade 3 (2), unknown grading (1)
Endocrine toxicities	*n* = 6 (hypothyroidism, hypophysitis)
Other	*n* = 3; lichen planus (1; grade 2), hyponatremia (1; grade 4), thrombocytopenia (1; grade 1)

## Data Availability

Anonymized data can be requested from the corresponding author if required. Institutional permission will need to be sought to share data with external parties.
